# Bioethanol from *Miscanthus* × *giganteus*: A Comparative Study of Different Pretreatment Technologies

**DOI:** 10.3390/polym18121551

**Published:** 2026-06-22

**Authors:** Ekaterina A. Skiba, Ekaterina I. Kashcheyeva, Vladimir N. Zolotukhin, Galina F. Mironova, Vera V. Budaeva

**Affiliations:** Bioconversion Laboratory, Institute for Problems of Chemical and Energetic Technologies, Siberian Branch of the Russian Academy of Sciences (IPCET SB RAS), Biysk 659322, Russia; eas08988@mail.ru (E.A.S.); massl@mail.ru (E.I.K.); zolo-i@mail.ru (V.N.Z.); yur_galina@mail.ru (G.F.M.)

**Keywords:** bioethanol, *Miscanthus* × *giganteus*, pretreatment, nitric acid, enzymatic hydrolysis, impurities, yield

## Abstract

Second-generation bioethanol technology is based on renewable raw materials with an unlimited potential for replenishment. However, the production cost of second-generation bioethanol is still higher than that of the first-generation. Biomass pretreatment is a key challenge, and solving it will improve the technology efficiency. In this study, *Miscanthus* × *giganteus* from the Russian breeding stock was subjected to pretreatments with dilute HNO_3_ under atmospheric pressure. Pretreatments were carried out either as a single stage (HNO_3_) or as two stages ((i) HNO_3_ followed by NaOH, and (ii) NaOH followed by HNO_3_). Classical delignification with NaOH was also performed for comparison. Simultaneous saccharification and fermentation with delayed inoculation (dSSF) was then performed under identical conditions, with *Saccharomyces cerevisiae* Y-3136 as the microbial producer. Two-stage pretreatments were found to excel in purity, pulp composition, pulp conversion, bioethanol yield during fermentation, and raw bioethanol purity (impurities decreased by a factor of 21 compared to NaOH delignification). However, fermentation indicators are not the only critical aspect in bioethanol production technology. The complete cycle from *Miscanthus* × *giganteus* feedstock to the target bioethanol product was evaluated. The single-stage pretreatment with HNO_3_ performed best among the tested conditions. The HNO_3_ pretreatment achieved a 50% yield of pulps and a maximal bioethanol yield of 267 L/t, which is 44% higher compared to NaOH delignification. Furthermore, the HNO_3_ pretreatment enables savings in resources and electric power, as well as full commercial utilization of all polymers of the lignocellulosic matrix of the feedstock.

## 1. Introduction

Given the depletion of fossil fuel reserves, the transition of multiple sectors of the global economy to biofuels is inevitable. Beyond addressing resource scarcity, biofuel production technology also contributes to environmental improvement [1,2]. Bioethanol is considered a carbon-neutral product due to the equivalent exchange of carbon during combustion and biomass formation [3,4,5]. The ethanol market is projected to grow from US$106 million per year in 2022 to US$178 million per year by 2030, with a cumulative annual growth rate of 6.73% [6].

As demonstrated by the latest review papers [1,7,8,9,10,11,12], the development of the second-generation bioethanol technology is a global priority. The first-generation bioethanol technology is the cheapest but it competes with food production. The third- and fourth-generation bioethanol technologies can be implemented mainly in subtropical climatic zones of the planet.

Second-generation bioethanol technology is based on exceptionally cheap non-food cellulosic feedstock, i.e., renewable raw materials with an unlimited potential for replenishment. Currently, experts worldwide agree that pretreatment determines the efficiency of the entire second-generation bioethanol technology [8,13,14,15,16,17]. The success of subsequent technological stages—enzymatic hydrolysis and alcoholic fermentation—depends on the pretreatment efficiency. The lignocellulosic matrix is highly recalcitrant and consists of polymers, such as cellulose, hemicelluloses, lignin, as well as mineral substances. Pretreatment is required to enhance the accessibility of these polymers to enzymatic action [18]. Pretreatment can be physical, chemical, biological, or combined [16,19]. According to Zabed et al. [13], a method should meet seven efficiency criteria for its industrial adoption: (1) high enzymatic hydrolysis efficiency; (2) low losses of hemicellulose and cellulose; (3) undesirable inhibitors must not be formed; (4) low energy consumtion; (5) low biomass losses; (6) reduction in byproducts or waste; (7) low consumption of chemical reagents. Zabed et al. [13] emphasize that none of the existing methods meets all aforesaid requirements, with each method having some drawbacks.

Saad and Gonçalves [20] point out that a number of industrial initiatives were unsuccessful due to the inefficient method chosen for pretreatment. Currently, pretreatment and enzymatic hydrolysis account for 40% of the cost in commercialized technologies [17]. Therefore, the search for new methods aimed at overcoming the aforesaid limitations is highly relevant.

The use of nitric-acid pretreatment of cellulosic feedstocks for subsequent enzymatic hydrolysis and bioethanol production has been described in the literature. For instance, nitric-acid pretreatment has been applied to rice straw [21], corn stover [22], corncobs [23], oil palm fronds [24], sugarcane bagasse [24,25,26,27], and *Miscanthus* [28]. Moreover, researchers unanimously note the advantage of nitric acid over sulfuric acid in terms of the enzymatic digestibility of the resultant substrates. However, in all of the listed studies, the nitric-acid pretreatment process was conducted at temperatures above 100 °C and at pressures above the atmospheric, which creates extremely hazardous production conditions and pose great problems for its adoption.

In the present study, we employed pretreatment methods for cellulosic biomass based on nitric-acid. These methods have two distinctive features: first, they are carried out at atmospheric pressure and, second, they use a dilute (4 wt%) nitric acid solution. Pretreatment was performed as a single stage (with acid or alkali) and as two stages (with the two reagents sequentially). NaOH delignification, a classical method for pretreatment of non-woody cellulosic raw materials [29,30,31], was used as the reference method because it is this method that is most often used for comparison [32].

The fundamental distinction of the present study is that the process was conducted under atmospheric pressure. Operating at atmospheric pressure provides safer and more environmentally friendly production conditions and reduces the formation of degradation products from the polymers of the lignocellulosic matrix into compounds with inhibitory properties. This is an important advantage from the technological standpoint, as it avoids the need for detoxification of inhibitors after pretreatment [14,17]. Moreover, Sanchez et al. [27] note that the higher the pressure during the pretreatment of feedstocks with nitric-acid, the greater the accumulation of fuel oils in the synthesized bioethanol. To the best of our knowledge, our research team is the first and currently the only one working with dilute nitric acid solutions under atmospheric pressure, as noted in the review paper [33].

Previously, we used the single-stage pretreatment with nitric acid to obtain bioethanol from annual raw materials, namely oat hulls [34]. In the present study, we used perennial *Miscanthus* × *giganteus* as the model cellulosic feedstock. *Miscanthus* is one of the world’s leading feedstocks for second-generation bioethanol production [28,35,36,37,38]. This grass has been widely introduced and is now found in regions with climates ranging from tropical to temperate [39].

The novelty of this work is the first comparative study of different pretreatment methods (2 single-stage and 2 two-stage methods) applied to the complete cycle of converting *Miscanthus* × *giganteus* into bioethanol. A thorough analysis was performed on the effect of pretreatment on the pulp composition, enzymatic hydrolysis and alcoholic fermentation processes, and finally, raw bioethanol quality. The latter issue is rarely raised in the literature and merits closer examination.

## 2. Materials and Methods

### 2.1. Feedstock

*Miscanthus* × *giganteus* (the KAMIS variety) from the Russian breeding stock was used herein for the first time as the feedstock for biotechnological conversion into bioethanol. *Miscanthus* × *giganteus* from a 7-year-old plantation (Marushkino village, the Moscow region, Russia) was kindly provided by OOO “Master Brand”.

### 2.2. Microbial Producer

Non-genetically modified yeast *Saccharomyces cerevisiae* Y-3136 (Russian National Collection of Industrial Microorganisms, Moscow, Russia) was utilized as the microbial producer.

### 2.3. Pretreatment

The feedstock was pretreated in one stage or two stages at atmospheric pressure, at 90–96 °C, at a hydromodulus of 1:20, under lab-scale conditions to obtain the following chemical pretreatment products:

(1) Method 1: Pretreatment of *Miscanthus* × *giganteus* in one stage with a 4 wt% nitric-acid solution for 8 h to yield pulp 1;
(2) Method 2: Pretreatment of *Miscanthus* × *giganteus* in one stage with a 4 wt% sodium hydroxide solution for 8 h to yield pulp 2;
(3) Method 3: Pretreatment of *Miscanthus* × *giganteus* in two stages with a 4 wt% nitric-acid solution for 10 h first and then, after water washing, with a 4 wt% sodium hydroxide solution for 2 h to yield pulp 3;
(4) Method 4: Pretreatment of *Miscanthus* × *giganteus* in two stages with a 4 wt% sodium hydroxide solution for 10 h first and then, after water washing, with a 4 wt% nitric-acid solution for 2 h to yield pulp 4.

The use of a 4 wt% nitric-acid solution was substantiated in our previous work [40].

The pulps were thoroughly washed with water. Wet pulps were used for the studies, with all weighed samples taken on absolutely dry basis. The calculation of the pulp yields is given in the Supplementary Materials.

The pulps differed fundamentally in the isolation methods. The reactivity to enzymatic hydrolysis, dSSF, bioethanol yield, and bioethanol impurities were studied under the same conditions.

### 2.4. Reactivity to Enzymatic Hydrolysis

Enzymatic hydrolysis was performed using a cocktail of enzymes Agrocell Plus (Agroferment LLC, Moscow, Russia), Cellolux-A (Sibbiofarm LLC, Berdsk, Russia), and Ultraflor Max (Novozymes A/S, Bagsværd, Denmark) (1:1:1). The substrate (4.5 g) and acetate buffer (150 mL, pH 4.7) were mixed in an Erlenmeyer flask, and the enzyme cocktail (20 mg enzyme/1 g substrate) was then added. The reaction was carried out on an EKROS PE-6410 horizontal mixing device (Echokhim LLC, Ufa, Russia) at a temperature of 46 ± 2 °C. To monitor the release of reducing sugars (RS), 3 mL aliquots were taken at 8 h intervals. The aliquots were sealed and incubated for 5 min in a boiling water bath to cease the hydrolysis process, then filtered, and the liquid phase was analyzed for the RS concentration on a glucose basis. After 72 h hydrolysis, the suspension was filtered in vacuo, and the concentrations of RS and pentose in the filtrate were analyzed.

The calculation of the final yields of reducing sugars (1) on a substrate weight basis and (2) on a hydrolyzables content basis is given in the Supplementary Materials.

### 2.5. Simultaneous Saccharification and Fermentation with Delayed Inoculation (dSSF)

Bioethanol was produced via simultaneous saccharification and fermentation with delayed inoculation (dSSF), as this method is recognized as the most efficient [41]. Enzymatic hydrolysis was carried out using the aforesaid enzyme cocktail at an initial solid loading of 60 g/L. After 24 h, the temperature was lowered to 28 °C, 10 wt% yeast inoculum was added, and dSSF was carried out for another 72 h, with a total dSSF process time of 96 h. The process was run in 2 L Erlenmeyer flasks placed into a Unimax-1010 incubated orbital shaker (Heidolph Instruments GmbH & Co. KG, Schwabach, Germany), with the working volume of the medium being 1 L.

The calculation of the bioethanol yield is given in the Supplementary Materials.

### 2.6. Analytical Methods

The compositions of the feedstock and pulps were quantified by classical wet methods [42]. The RS concentration on a glucose basis was measured spectrophotometrically using a reagent based on 3,5-dinitrosalicylic acid (Panreac, Castellar del Vallès, Spain) [43]. The concentration of pentoses on a xylose basis was quantified spectrophotometrically using an orcin solution [44]. A UNICO UV-2804 spectrophotometer (United Products and Instruments, Inc., Dayton, NJ, USA) was employed. pH was determined potentiometrically. The yeast count was quantified by the direct-count method in a Goryaev chamber [45]. The concentration of bioethanol in the mashes was measured areometrically after direct distillation. The compositional profile of raw bioethanol was determined using gas–liquid chromatography [46]. A gas chromatograph with a Crystal 2000M flame ionization detector (SKB Chromatec, Yoshkar-Ola, Russia) was used with a ZB-FFAP capillary gas chromatography column (Phenomenex, Torrance, CA, USA).

All reagents were purchased from AO Vecton (Saint-Petersburg, Russia). The experimental results were obtained in triplicate and statistically processed by standard methods using Microsoft Office Excel 2019 software.

## 3. Results and Discussion

### 3.1. Pretreatment of Miscanthus × giganteus

**Figure 1:**
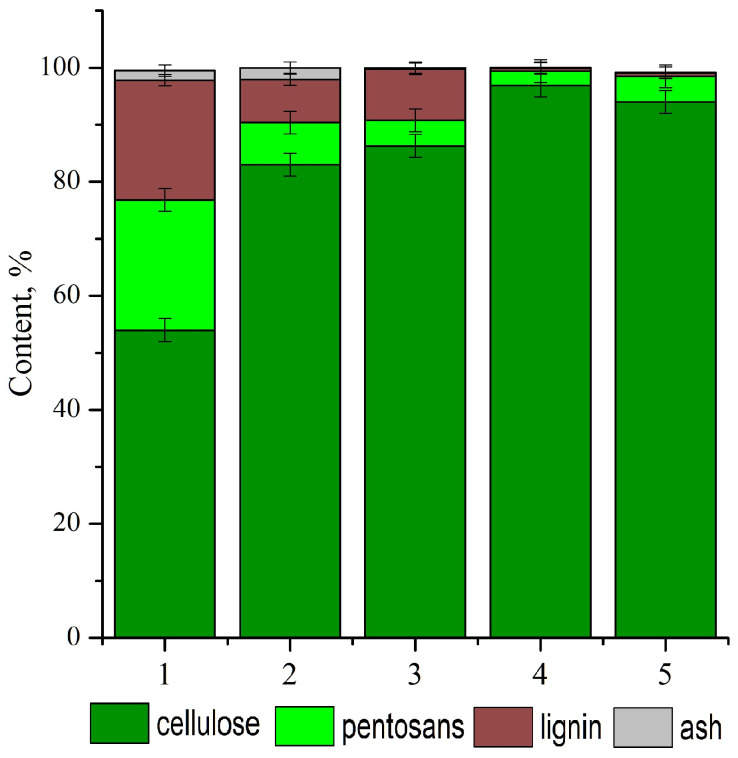
Chemical composition of *Miscanthus* × *giganteus* and its pulps. Error bars represent the standard deviation.

The chemical composition of *Miscanthus* × *giganteus* and its pulps is shown in Figure 1.

The pulp composition was dependent on the reagent (nitric acid and sodium hydroxide) and the number of chemical pretreatment stages. During pretreatment with nitric acid, the cellulose content in pulp 1 increased 1.5-fold (from 54 ± 0.4% in *Miscanthus* × *giganteus* to 83 ± 0.4%). At the same time, the proportion of non-cellulosics declined: pentosans by 3.0 times (from 22.8 ± 0.1% in the feedstock to 7.4 ± 0.1%) and lignin by 2.8 times (from 21.0 ± 0.1% in the feedstock to 7.5 ± 0.1%). On the other hand, the proportion of mineral components, i.e., ash content, rose 1.2-fold (from 1.70 ± 0.01% in the feedstock to 2.10 ± 0.01%), which is peculiar to the action of nitric acid. A similar dependence was obtained when oat hulls were pretreated with nitric acid [47]. The chemical composition of pulp 1 suggested that enzymatic hydrolysis would be successful, since total hydrolyzables increased 1.2-fold (from 76.8 ± 0.4% in the feedstock to 90.4 ± 0.4%).

Pretreatment with sodium hydroxide provided a 1.6-fold increase in the cellulose content in pulp 2 and a reduction in all non-cellulosics: pentosans by 5.1 times, lignin by 2.3 times, and ash by 8.9 times. As a result, total hydrolyzables increased 1.2-fold compared to untreated *Miscanthus* × *giganteus*. By comparing the methods, let us formally note that the pretreatment with nitric acid enables a 1.2-fold increment in the proportion of hydrolyzables, just like alkaline delignification; that is, one can assume that they have equal reactivity to enzymatic hydrolysis. It is always emphasized that alkaline delignification allows for the effective removal of lignin from the lignocellulosic matrix, but one can see that the pretreatment with nitric acid demonstrated a more outstanding result: more specifically, pulp 1 contained 7.5 ± 0.1% acid-insoluble lignin, while pulp 2 contained 9.0 ± 0.1%. Our results significantly exceed those reported [48], where the achieved mass contents of cellulose in the pulps were 66% after sulfuric acid pretreatment and 70% after alkaline pretreatment.

The two-stage pretreatment of *Miscanthus* × *giganteus* yielded very pure pulps 3 and 4. The pulps contained 96.9 ± 0.4% and 94.0 ± 0.4% cellulose, respectively, and total hydrolyzables accounted for 99.4 ± 0.4% and 98.3 ± 0.4%, respectively. It could be assumed that they had a very high reactivity to enzymatic hydrolysis.

Table 1 presents the calculated conversion of the major polymers into pulps depending on their content in the feedstock (*Miscanthus* × *giganteus*).

The single-stage methods (Methods 1 and 2) enable the maximal extraction of cellulose, providing at least an 80% removal of non-cellulosic impurities—pentosans and lignin. The pulp yield on a *Miscanthus* × *giganteus* weight basis is approximately 50%.

The two-stage methods enable sufficiently complete removal of pentosans and lignin, leaving behind only 0.8–7.5% in the pulps. However, under these conditions, significant hydrolysis of cellulose also occurs: only 57–66% of cellulose transfers to the pulps, and the pulp yield is only 32–38%.

Thus, the cost of obtaining chemically pure pulps is very high: the yield of pulps 3 and 4 derived by the two-stage methods decreases by 21–36% compared to the pulps obtained via the single-stage methods.

### 3.2. Determination of Reactivity to Enzymatic Hydrolysis

The dSSF process was applied to produce bioethanol, in which it is impossible to determine how much sugars have accumulated as a result of enzymatic hydrolysis, because the bioethanol biosynthesis begins to consume sugars immediately. However, it is extremely important to assess the convertibility of substrates in order to understand which pretreatment method is the most efficient. Therefore, we first examined the reactivity to enzymatic hydrolysis.

**Figure 2:**
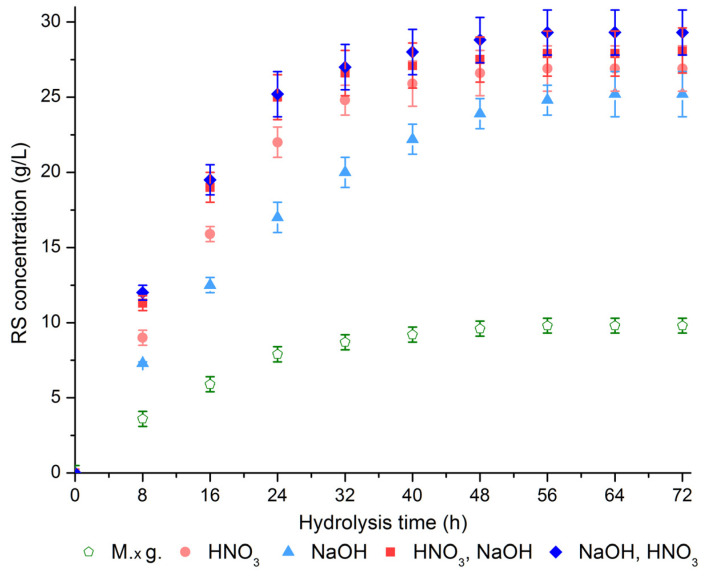
Concentration of reducing sugars (RS) plotted against enzymatic hydrolysis time of *Miscanthus* × *giganteus* (M. × g.) and its pulps. Error bars represent the standard deviation.

The enzymatic hydrolysis efficiency of *Miscanthus* × *giganteus* before and after pretreatment is presented in Figure 2 and Table 2.

The 72 h hydrolysis of untreated *Miscanthus* × *giganteus* resulted in the release of 9.8 g/L RS, in which case the cellulose conversion rate was 54%, the conversion rate of hydrolyzable constituents of the substrate was 38%, and the yield was 29% on a substrate weight basis. As expected, *Miscanthus* × *giganteus* prior to treatment demonstrated the lowest enzymatic hydrolysis efficiency among the substrates studied. Untreated *Miscanthus* × *giganteus* is poorly amenable to enzymatic hydrolysis. This is explained by the properties of the pristine feedstock, including high crystallinity, low porosity, and high contents of lignin and hemicellulose [49].

In the study by Lee et al. [49], the cellulose conversion rate during the hydrolysis of untreated *Miscanthus sacchariflorus* was less than 20% (which is 2.7 times lower than in the present study). Xu et al. [50] were able to achieve a reducing sugar yield of 9% from untreated *Miscanthus sinensis* (which is 4.2 times lower than in the present study). Świątek et al. [51] confirmed the poor reactivity of untreated *Miscanthus* × *giganteus* to enzymatic hydrolysis, but the use of the highly efficient enzyme cocktail made it possible to achieve a glucose yield of 25.6% on a cellulose content basis (which is 2.1 times lower than in the present study). The RS yield obtained herein by hydrolysis of *Miscanthus* × *giganteus* exceeds those reported in the literature and can be explained solely by the high efficiency of the enzyme mixture used. In this case, a leading role was played here by the Agrocell Plus enzyme, which is characterized by a high content of endoglucanases (about 50% of all proteins) and, accordingly, a high CMCase activity (48 U/mg protein), as shown by Semenova et al. [52].

Pretreatment of *Miscanthus* × *giganteus* provided a 2.6–3.0-fold increase in the efficiency of enzymatic hydrolysis. In this case, the reactivity of the substrates followed the sequence: 4 > 3 > 1 > 2 > M. × g. The yield of reducing sugars on a pulp weight basis, or convertibility, varied from 76 to 81%, which closely correlates with the literature results [28,38].

The concentration of pentoses on a xylose basis in all hydrolyzates derived from pulps allows the hydrolyzates to be called glucosic: the contribution of xylose to the total RS concentration was 2–7%. The low xylose content is a positive factor, since *Saccharomyces cerevisiae*, which is unable to ferment pentoses, is most often used as the microbial producer for bioethanol synthesis [35,37,38]. In the present study, we also adhered to the commonly accepted methodology to ensure the reproducibility of scientific results and facilitate their comparability.

### 3.3. dSSF of Miscanthus × giganteus Pulps

Simultaneous saccharification and fermentation with delayed inoculation (dSSF) is used for conducting non-isothermal processes of enzymatic hydrolysis and alcoholic fermentation. Glucose resulting from enzymatic hydrolysis during dSSF is almost immediately converted into ethanol by microorganisms, thus preventing inhibition of enzymatic hydrolysis. The advantages of dSSF also include a reduced risk of contamination, lower energy inputs, capital and labor costs, and an enhanced bioethanol yield [53]. This is precisely why dSSF was chosen in the present work.

**Figure 3:**
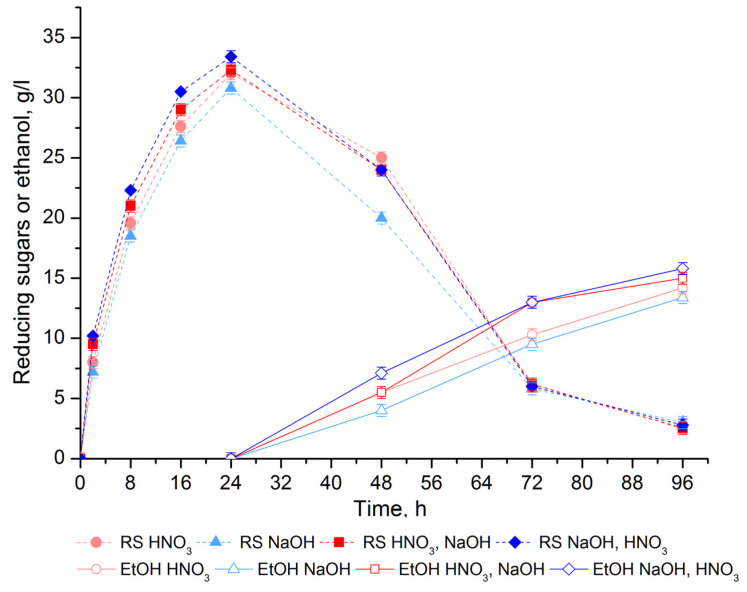
Time course of concentrations of reducing sugars (RS) and bioethanol (et) during delayed simultaneous saccharification and fermentation (dSSF). Error bars represent the standard deviation from triplicate experiments.

The concentration of reducing sugars plotted against the dSSF process time is displayed in Figure 3.

Enzymatic hydrolysis was carried out for initial 24 h; therefore, the concentration of reducing sugars was increasing. During this period of concentration increment, the pulps could be arranged in the following order: 4 > 3 > 1 > 2. After 24 h, the concentration of reducing sugars ranged from 30.8 g/L for pulp 2, a substrate with the lowest convertibility, to 33.4 g/L for pulp 4, a substrate with the highest convertibility. The reduced reactivity of pulp 2 to enzymatic hydrolysis seems to be associated with its high residual lignin content. It has previously been shown that the single-stage treatment of oat hulls with nitric acid produces a pulp being a high-quality substrate for enzymatic hydrolysis. A unique feature of nitric acid is that it is able to nitrate lignin, and the nitrated acid-insoluble lignin in the substrate is no longer inhibitory to enzymatic hydrolysis [47]. The present study with *Miscanthus* × *giganteus* proves that, in terms of reactivity to enzymatic hydrolysis, pulp 1 obtained by pretreatment with nitric acid has advantages over pulp 2 derived by alkaline delignification.

To summarize, our forecast regarding the reactivity to enzymatic hydrolysis held true: the purer the pulp, the higher the reactivity to enzymatic hydrolysis. However, this increase is overall insignificant: for example, after 24 h, the concentration of reducing sugars for pulp 3 was only 0.3 g/L higher than for pulp 1, so the question of the expediency of the two-stage pretreatment at the enzymatic hydrolysis stage remains open.

Traditionally, binary sequential pretreatment with acids and alkali is used to remove the hemicellulose fraction (using acids) and the lignin fraction (using alkalis) to obtain reference-grade pure substrates for enzymatic hydrolysis [54], since it is classically believed that both hemicellulose and, even more so, lignin hinder enzymatic hydrolysis [15]. In our case, the purest pulp 3 exhibited lower hydrolyzability than pulp 4, which is explained by the high cellulose crystallinity of pulp 3. Obtaining an especially pure pulp is conjugated with a decrease in its yield, in which case hydrolysis of the amorphous part of cellulose occurs since the yield was 32% for pulp 3 and 38% for pulp 4 on a *Miscanthus* weight basis (Table 3). Similar instances of the lowered reactivity to enzymatic hydrolysis due to the increased cellulose crystallinity are described in the literature [48,55].

**Figure 4:**
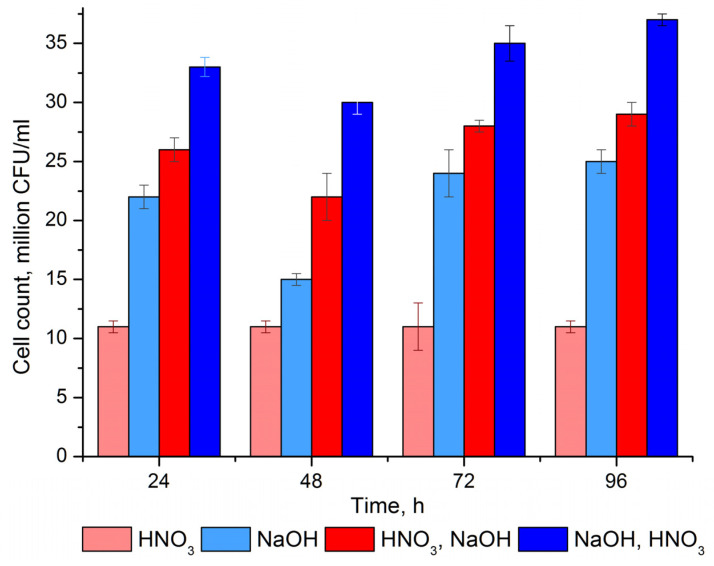
The yeast count depending on the pretreatment method of *Miscanthus* × *giganteus*. Error bars represent the standard deviation.

In terms of the bioethanol concentration in the mashes, the pulps can be ranked as follows: 4 > 3 > 1 > 2, i.e., the one-stage pretreatment methods of *Miscanthus* at the fermentation stage proved to be less effective than the two-stage ones. For the single-stage pulps 1 and 2, the final concentrations of bioethanol in the mashes were 14.2 g/L and 13.4 g/L, respectively, whereas the final concentrations for the two-stage pulps 3 and 4 were 15.0 g/L and 15.8 g/L, respectively. This is explained by the biologically good quality of the nutrient media prepared from the pulps pretreated in two stages. The pulps devoid of non-cellulosic impurities when hydrolyzed gave cleaner nutrient media on which the yeast proliferated better, as can be seen in Figure 4.

At yeast inoculation (24 h after dSSF initiation), the yeast counts were equivalent across all pulp substrates. By 48 h, the pulps could be ranked by the yeast count in the descending order: 4 > 3 > 1 > 2. This pattern persisted until the end of the process and corresponded to the ranges of the bioethanol concentrations in the mashes. The lagging performance of pulp 2 can be explained by the lower concentrations of reducing sugars in the nutrient medium prepared from pulp 2 as compared to the other substrates; in addition, the residual sodium ions in the medium had an adverse effect [34].

### 3.4. Compositional Profile of Impurities in Experimental Raw Bioethanol Samples from Miscanthus × giganteus

The compositional profile of impurities in the raw bioethanol samples strongly depends on the pretreatment method of *Miscanthus* × *giganteus* (Table 3).

In raw bioethanol obtained from pulp 1, the main impurities were fuel oils (791 mg/dm^3^ isobutanol > 285 mg/dm^3^ isoamylol > 156 mg/dm^3^ 1-propanol) and acetaldehyde (510 mg/dm^3^). The total impurity content was 1.34 wt%.

Among all raw bioethanol samples, the sample from pulp 2 had the highest amount of impurities. The sample contained not only much fuel oils (1170 mg/dm^3^ 1-propanol > 983 mg/dm^3^ isoamylol > 806 mg/dm^3^ isobutanol), but also 8850 mg/dm^3^ acetaldehyde and esters (2654 mg/dm^3^ ethyl acetate > 728 mg/dm^3^ methyl acetate). The total impurity content was 6.43 wt%, which is five times higher than in raw bioethanol from pulp 1. At the same time, bear in mind that the total hydrolyzables were the same for pulps 1 and 2.

The two-stage pretreatment used for pulps 3 and 4 yielded much purer raw bioethanol samples than the single-stage one. Thus, the content of fusel oils decreased ten- and eightfold, the content of acetaldehyde decreased 56- and 25-fold, the content of esters decreased 1990- and 1692-fold, and the total impurity content decreased 21- and 18-fold, respectively, as compared to raw bioethanol from pulp 2 obtained by the classical method. The purest raw bioethanol was obtained from pulp 3 (the total impurity content was only 0.3%), and it was this pulp that was the purest after pretreatment. These arguments are indicative of the advantage of the two-stage methods of the feedstock pretreatment in the complete cycle of bioethanol production.

Thus, two parameters affected the purity of raw bioethanol samples. First, the number of pretreatment stages: the two-stage pretreatment reduced the total amount of impurities in raw bioethanol by 4–21 times compared to the single-stage pretreatment. Second, the single-stage pretreatment method, such as pretreatment with nitric acid, allowed for the production of raw bioethanol that is five times purer than that produced by the classic alkaline delignification.

The issue regarding the quantity and composition of impurities in raw bioethanol is rarely discussed in the literature. On one hand, this is quite explainable, since modern purification methods allow for the easy removal of impurities from bioethanol [56], and the separated impurities find wide application in the chemical industry [57]. On the other hand, the fundamental question regarding impurities in raw bioethanol is necessary for the technology, the answer to which allows finding strategic solutions in the complete bioethanol cycle. It is known that the composition and quantity of impurities in raw bioethanol allows for identifying the origin of bioethanol and detecting the process failures and unfavorable conditions for the vital activity of yeast [58,59].

It is noteworthy that the raw bioethanol samples presented in this study had an extremely low methanol content ranging from 0.001 wt% in the purest sample prepared from pulp 3 to 0.007 wt% in the sample from pulp 2 obtained by alkaline delignification. This is due to the use of non-food, non-woody feedstock, since methanol originates either from pectin substances in food-grade raw materials or from glucuronoxylan and arabinoglucuronoxylan in hardwood raw materials. In addition, methanol can originate from lignin under harsh process conditions [59]. In our case, mild pretreatment modes were applied at atmospheric pressure. The small amount of methanol in pulp 2 is likely due to the use of alkaline delignification. In pulp 1, lignin underwent oxidative nitration, so there was practically no methanol in the pulp 1 sample. Pulps 3 and 4 were very clean, so methanol formation was extremely low. The methanol content in the experimental samples was thousand times lower than that described in the review paper by Habe et al. [58].

In this study, the total impurity content in raw bioethanol from pulp 1 was five times as low as that in raw bioethanol from pulp 2. This is consistent with the data from [34], where a similar dependence was observed when bioethanol was derived from oat hulls: the total amount of impurities was 0.49 wt% when the feedstock was treated with nitric acid and 3.66% when the feedstock was treated by alkaline delignification. The increase in the amount of impurities in the raw bioethanol samples from *Miscanthus* × *giganteus* indicates the presence of inhibitors in *Miscanthus* itself, which is explained by the nature of this fast-growing biomass and is well described in the literature [28,60].

One of the important markers of bioethanol quality is the content of fuel oils (among the main ones are 1-propanol, 2-methyl-1-propanol, and 3-methyl-1-butanol). In the study by Sanchez et al. [61], the content of fusel alcohols in raw bioethanol samples ranged from 223 to 547 mg/dm^3^, which is consistent with our study results for pulp 1. That said, those authors conclude that the use of nitric acid under optimal conditions for pretreating sugarcane press-mud is preferable to hydrothermal pretreatment. Sanchez et al. [27] showed that the contents of bioethanol and fusel oils were directly proportional, but using the Box–Behnken design method made it possible to find optimal conditions that reduce the content of impurities in bioethanol. Fusel alcohols are known to originate during fermentation through either (i) the Ehrlich metabolic pathway or (ii) the de novo synthesis pathway [62]. The excessive formation of fusel oils in the case of pulp 2 indicates unfavorable conditions for the yeast growth. We consider the presence of sodium ions in the nutrient medium to be the key negative factor [34].

Thus, the one-stage pretreatment of *Miscanthus* × *giganteus* with nitric acid allows for the synthesis of purer raw bioethanol compared to alkaline delignification. This is an important technological advantage of using nitric acid in the complete cycle of bioethanol production from *Miscanthus* × *giganteus*.

### 3.5. Bioethanol Yield from Miscanthus × giganteus and Comparison with the Global State of the Art

In terms of the compositional purity, bioethanol yield and purity of raw bioethanol, the two-stage pretreatments of *Miscanthus* × *giganteus* were the best, but the main criterion for production efficiency is the yield of the end product per unit of feedstock mass. The calculation of bioethanol yield is given in Table 4.

In terms of the bioethanol yield from 1 ton of *Miscanthus*, the pretreatment methods that provide the maximal bioethanol yield can be ranked as follows: 1 > 2 > 4 > 3. The difference between the first and last values is 30%. The leader in terms of the maximal bioethanol yield was the single-stage pretreatment with a dilute nitric-acid solution (preparation of pulp 1).

The two-stage treatment led to an increase in the pulp purity due to a decrease in its yield, which is a critical factor. In addition, it should be noted that the two-stage treatment led to a twofold increase in the consumption of reagents and water for pulp washing, to an increase in energy inputs and working time, and, ultimately, to an increase in the cost of bioethanol.

*Miscanthus* × *giganteus* is one of the world’s leading feedstocks for bioethanol. Our bioethanol yields are consistent with the global data (Table 5). The bioethanol yield obtained by the nitric-acid treatment of *Miscanthus* × *giganteus* exceeds those described in [35,38] by 2.0 and 1.8 times, respectively, though we used solid loadings of 1.7 and 2.1 times lower, respectively, than those in the cited literature sources, indicating the effectiveness of the nitric-acid pretreatment. The yield we obtained exceeds that achieved by Cerazy-Waliszewska et al. [37], despite the fact that the initial substrate loading in our work was tenfold lower than that in [37]. Apparently, 625 g/L is an excessive initial substrate loading at which mass-exchange processes are impeded [63].

The yield achieved in this work is on par with that achieved by Zhang et al. [36], who used a high initial substrate concentration of 170–200 g/L. This concentration appears to be close to optimal for highly concentrated substrates. To maximize the bioethanol yield in the future, we plan to apply an initial substrate loading of 200 g/L for nitric-acid-pretreated *Miscanthus* × *giganteus*, implementing a short-time feeding strategy during high-solid hydrolysis [64].

### 3.6. Benefits of Nitric-Acid Pretreatment at Atmospheric Pressure

The pretreatment with nitric acid at atmospheric pressure is a method that can be successfully commercialized for the following reasons: first, it has demonstrated good scalability in practice [34]; second, the spent nitric-acid solution can be used tenfold without reduction in the reactivity of the resulting substrates to enzymatic hydrolysis [65], making the use of chemicals economical; thirdly, our previous work [66] demonstrated that nitric-acid treatment induces artificial humification of the lignocellulosic matrix components. As a result, the spent liquor can successfully be repurposed as a plant fertilizer and growth stimulant. This indicates that the method is not only integrated, but also environmentally friendly.

The key criteria for an effective pretreatment were outlined by Zabed et al. [13]. The one-stage nitric-acid pretreatment used in this work fulfills most of these criteria and ensures: (1) high efficiency of enzymatic hydrolysis; (2) savings in reagents and energy through its single-stage design; (3) low consumption of chemical reagents and energy due to the tenfold reuse of the spent nitric-acid solution; (4) no need for detoxification of the media, as this method provides good biological quality of the hydrolyzates and reduces the concentration of impurities in the raw bioethanol; (5) operation at atmospheric pressure, which significantly lowers energy inputs and enhances process safety; (6) holistic utilization of all lignocellulosic components, as the cellulose fraction is converted into bioethanol, while the hemicellulose and lignin fractions are valorized into a fertilizer; and (7) good process scalability. Therefore, it is expected that the single-stage nitric-acid pretreatment technology can be commercially successful for bioethanol production from *Miscanthus* × *giganteus*.

### 3.7. Limitations and Solutions—Future Perspectives

In this work, four pretreatment methods of *M.* × *giganteus* were examined. The single-stage nitric-acid pretreatment is superior in bioethanol yield, while the two-stage pretreatment methods excel in bioethanol quality. The purity of raw bioethanol is an interesting scientific finding; however, performing two-stage pretreatment in order to obtain pure raw bioethanol is completely impractical because current purification techniques allow effective removal of bioethanol impurities.

The single-stage nitric-acid pretreatment significantly surpasses classical alkaline delignification by 1.2 times in terms of bioethanol yield from 1 ton of *M.* × *giganteus* and by 4.8 times in terms of raw-bioethanol purity. Furthermore, the single-stage nitric-acid pretreatment ensures the comprehensive utilization of all components of the lignocellulosic matrix.

Limitations of the use of single-stage nitric-acid pretreatment may be related to the morphology of the lignocellulosic matrix. It is known that the use of nitric acid for wood raw materials is not as effective as sulfate and sulfite pulping, which is due to the nature of wood lignin. The use of bast raw materials and their wastes (shives) also raises questions. Bast raw materials are known to be very recalcitrant to chemical, enzymatic, and microbiological treatments. Therefore, the issue regarding the universality of nitric-acid pretreatment of lignocellulosic biomass for the transformation into bioethanol remains open. The second limitation of using single-stage nitric-acid pretreatment is the high corrosion burden and potential hazard of the process, which necessitates the use of expensive, corrosion-resistant equipment. Running the process at atmospheric pressure reduces the production hazard. Since the spent solution is reused ten times, after which it can be used as a humic fertilizer [66], this method is sufficiently green. We also plan to perform a life cycle assessment of bioethanol production from *M.* × *giganteus*, similarly to that performed in [67].

To further enhance the bioethanol yield from *M.* × *giganteus*, we envision the following directions: first, integrated production of enzyme preparations and conversion of cellulose into C6 sugars within a single biorefinery [68]; second, the use of fed-batch enzymatic hydrolysis, which will enhance the concentration of C6 sugars in nutrient media to give a higher bioethanol yield [63]; third, the application of more efficient microbial producers like those obtained by genetic engineering methods [64]; and fourth, the conversion of stillage into biogas or feed premixes [69,70]. Taken together, these solutions would increase the efficiency of bioethanol production from *M.* × *giganteus*, reduce production costs, obtain additional products, and improve the environmental performance of the overall process.

## 4. Conclusions

*Miscanthus* × *giganteus* pretreatment methods performed as either one-stage or two-stages at atmospheric pressure using a dilute nitric-acid solution have been evaluated and compared with classical alkaline delignification of non-woody feedstocks for the first time.

Analysis of the complete conversion cycle of *Miscanthus* × *giganteus* into bioethanol clearly revealed the priority of the single-stage nitric-acid pretreatment compared to alkaline delignification and two-stage nitric-acid pretreatment methods. The one-stage pretreatment with nitric acid provided a high pulp yield of 50% (1.1–1.6 times higher than for the other methods), a high pulp convertibility of 81%, a fivefold reduction in impurities in raw bioethanol compared to alkaline delignification, and a high bioethanol yield of 267 L per ton of *Miscanthus* × *giganteus* (which is 17–44% higher than for the other methods).

This study was also focused on the analysis of impurities in raw bioethanol. Overall, the purer the pulp, the fewer impurities are found in raw bioethanol. For instance, the impurity content in the raw bioethanol samples produced via the two-stage pretreatment was 0.30–0.36 wt%, which is 18–21 times lower than that achieved with alkaline delignification. The distinctive feature of all raw bioethanol samples is that they are extremely low in methanol (0.001–0.007 wt%), which is explained by the pretreatments being conducted at atmospheric pressure.

The findings suggest that the single-stage nitric-acid pretreatment technology is expected to be commercially viable for bioethanol production from *Miscanthus* × *giganteus*.

## Figures and Tables

**Table 1:** Extraction degree of feedstock components into pulps.

Pulp (Method)	Yield on a *Miscanthus* Weight Basis, %	Extraction Degree of Component into Pulp, %			
		Kürschner Cellulose	Pentosans	Acid-Insoluble Lignin	Ash
1	50	76.9	16.2	17.9	61.8
2	48	76.7	9.5	20.6	5.4
3	32	57.4	3.5	0.8	2.3
4	38	66.1	7.5	1.1	1.3

**Table 2:** Enzymatic hydrolysis results for *Miscanthus* × *giganteus* and its pulps.

Characteristics of Hydrolyzate	M. × g.	1	2	3	4
RS:					
Concentration, g/L	9.8 ± 0.5	26.9 ± 0.5	25.2 ± 0.5	28.1 ± 0.5	29.3 ± 0.5
Yield on a substrate weight basis, %	29 ± 2	81 ± 2	76 ± 2	84 ± 2	88 ± 2
Yield on a hydrolyzables content basis, %	38 ± 2	89 ± 2	83 ± 2	85 ± 2	88 ± 2
Concentration of pentoses, g/L	5.8 ± 0.2	2.0 ± 0.2	1.1 ± 0.2	0.5 ± 0.2	0.9 ± 0.2

**Table 3:** Compositional profile of impurities in raw bioethanol samples.

Constituent	1	2	3	4	Unit of Measure
Acetaldehyde	510 ± 180	8850 ± 315	158 ± 12	357 ± 24	mg/dm^3^
Methyl acetate	8 ± 1	728 ± 12	1.0 ± 0.5	1.0 ± 0.5	mg/dm^3^
Ethyl acetate	31 ± 2	2654 ± 213	1.0 ± 0.5	1.0 ± 0.5	mg/dm^3^
Methanol	0.003 ± 0.001	0.007 ± 0.001	0.001 ± 0.001	0.002 ± 0.001	mg/dm^3^
1-Propanol	156 ± 14	1170 ± 144	28 ± 6	39 ± 7	mg/dm^3^
Isobutanol	791 ± 109	806 ± 204	1.0 ± 0.5	1.0 ± 0.5	mg/dm^3^
1-Butanol	72 ± 8	85 ± 7	143 ± 11	168 ± 13	mg/dm^3^
Isoamylol	285 ± 54	983 ± 106	2 ± 1	5 ± 1	mg/dm^3^
1-Pentanol	5 ± 1	7 ± 1	123 ± 12	138 ± 11	mg/dm^3^
Hexanol	1.0 ± 0.5	2 ± 1	6 ± 1	4 ± 1	mg/dm^3^
Total content of impurities	1.34 ± 0.05	6.43 ± 0.05	0.30 ± 0.05	0.36 ± 0.05	wt%

The concentrations of the constituents are given on an anhydrous alcohol basis.

**Table 4:** Calculation of bioethanol yield from 1 ton of *Miscanthus* × *giganteus*.

Indicators	Pulp (Method)			
	1	2	3	4
Pulp yield on a *Miscanthus* weight basis, %	50 ± 3	48 ± 3	32 ± 3	38 ± 3
Bioethanol yield on a pulp weight basis, wt%	53.5 ± 1	46.5 ± 1	58.1 ± 1	60.4 ± 1
Bioethanol yield from 1 ton of *Miscanthus*, kg	211 ± 5	176 ± 5	147 ± 5	181 ± 5
Bioethanol yield from 1 ton of *Miscanthus*, L	267 ± 5	223 ± 5	186 ± 5	229 ± 5

**Table 5:** Comparison of bioethanol yields from *Miscanthus* × *giganteus* obtained by different techniques.

Pretreatment	Solid Loading, g/L	Microbial Producer	Ethanol Concentration, g·L^−1^	Ethanol Yield from 1 t of *M.* × *giganteus*, L	Ref.
0.1 g/L NaOH, 121 °C	100	*S. cerevisiae* AS4	15.0–19.4	105–134	[35]
Ammonia fiber expansion	170–200	*S. cerevisiae* Y128 or *Zymomonas mobilis* 8b	33.7–38.0	252–284	[36]
1.5% NaOH, 90 °C, 5 h	625	*S. cerevisiae*, Lesaffre Advanced Fermentations	24.0–28.9	185–222	[37]
1% H_2_SO_4_, 121 °C, 30 min	125	*S. cerevisiae* NCYC2592	13.6	148	[38]
4 wt% HNO_3_, 94–96 °C, 6 h	60	*S. cerevisiae* Y-1693	18.1	267	Present paper

## Data Availability

The data that support the findings of this study are available on request from the corresponding author.

## Supplementary Materials

The following supporting information can be downloaded at https://www.mdpi.com/article/10.3390/polym18121551/s1, Formulas for calculation of the yields of intermediates and products.
