# Supplementary material for: Bioethanol from Miscanthus × giganteus: A Comparative Study of Different Pretreatment Technologies
Source: Polymers (Basel). 2026 Jun 22;18(12):1551. doi: 10.3390/polym18121551 (PMC13307000; doi:10.3390/polym18121551)
Supplement: Supplementary file 1 [file polymers-18-01551-s001.zip › polymers-4345054-supplementary.pdf]

Supplementary Materials

Bioethanol from *Miscanthus x giganteus*: A Comparative Study of Different Pretreatment Technologies

Ekaterina A. Skiba, Ekaterina I. Kashcheyeva, Vladimir N. Zolotukhin, Galina F. Mironova, and Vera V. Budaeva*

Bioconversion Laboratory, Institute for Problems of Chemical and Energetic Technologies, Siberian Branch of the Russian Academy of Sciences (IPCET SB RAS), Biysk 659322, Russia

**Correspondence: budaeva@ipcet.ru (Vera V. Budaeva)*

Calculations of the yields of intermediates and products on an oven-dry basis

The yield of each substrate on a feedstock weight basis was calculated by the formula:

$$\eta_{sub} = \frac{m_{sub}}{m_m} \cdot 100\ \%, \qquad \text{(S1)}$$

where η_{sub} is the substrate yield from the feedstock, %;

m_{sub} is the substrate weight after pretreatment, kg;

m_m is the *Miscanthus* weight, kg.

The final yields of reducing sugars (RS) (S2) on a substrate weight basis (convertibility) and (S3) on a hydrolyzables content basis were estimated by the formulas:

$$\eta_C = \frac{C_F \cdot V}{m_S} \cdot 0.9 \cdot 100 \qquad \text{(S2)}$$

$$\eta_H = \frac{C_F \cdot V}{m_S \cdot (100 - L - A)} \cdot 0.9 \cdot 100 \qquad \text{(S3)}$$

where η_C is the RS yield on a substrate weight basis, %;

η_H, is the RS yield on a basis of the hydrolyzables content in the substrate (convertibility), %;

C_F is the final RS concentration in the hydrolyzate, g/L;

V is the hydrolyzate volume, L;

0.90 is the coefficient attributed to the water molecule addition to anhydroglucose residues of the corresponding monomer units as a result of enzymatic hydrolysis;

m_S is the substrate weight for enzymatic hydrolysis, g;

L is the mass content of residual lignin in the substrate, %;

A is the mass content of ash in the substrate, %.

The yield of bioethanol on an RS concentration basis was calculated by the formula:

$$Y_{be} = \frac{C}{C_{RS} \cdot 0.6479} \cdot 100\ \%, \quad \text{(S4)}$$

where Y_{be} is the bioethanol yield, %;

C is the bioethanol concentration, vol.%;

C_{RS} is the RS concentration in the hydrolyzate, g/L;

0.6479 is the conversion factor of glucose into ethanol.

The total yield of bioethanol on a *Miscanthus* weight basis was calculated by the formula:

$$\eta_{BE} = \frac{\eta_{Sub} \cdot \eta_{RS} \cdot \eta_{be}}{10000}, \quad \text{(S5)}$$

where η_{BE} is the total yield of bioethanol on a *Miscanthus* weight basis, %;

η_{sub} is the substrate yield from the feedstock, %;

η_{RS} is the RS yield on a substrate weight basis, %;

η_{be} is the bioethanol yield on an RS concentration basis, %.
